# Study on the Dynamic Changes in Synaptic Vesicle-Associated Protein and Axonal Transport Protein Combined with LPS Neuroinflammation Model

**DOI:** 10.1155/2013/496079

**Published:** 2013-09-24

**Authors:** Rui Zhang, Ming Zhao, Hai-jie Ji, Yu-he Yuan, Nai-hong Chen

**Affiliations:** State Key Laboratory of Bioactive Substances and Functions of Natural Medicines, Institute of Materia Medica, Chinese Academy of Medical Sciences and Peking Union Medical College, Beijing 100050, China

## Abstract

Microglia activation is the major component of inflammation that constitutes the characteristic of neurodegenerative disease. A large amount of researches have demonstrated that inflammation involved in the pathogenesis of PD process activated microglia acting on the neurons through the release of a variety of inflammatory factors. However, the molecular mechanism underlying how it does work on neurons is still unclear. Here, we show that intracerebral injections of LPS induced Parkinson's disease pathology in C57BL/6J mice. Furthermore, study on the dynamic changes in Synaptic vesicle-associated protein and axonal transport Protein in this process. The results indicated that after administration of LPS in the brain, the inflammatory levels of TNF-**α** and IL-1**β** both are elevated, and have a time-dependent.

## 1. Introduction

Parkinson's disease (PD) is primarily an age-related debilitating neurodegenerative disorder characterized by a selective and gradual loss of dopaminergic (DA) innervations from the substantia nigra pars compacta (SNpc) to the striatum (caudate and putamen) of the basal ganglia [1, 2]. Progressive degeneration of the nigrostriatal DA pathway eventually leads to the development of clinical symptoms that include bradykinesia, rigidity, tremor, and defective gait, mostly in people over the age of 60 [3]. Postmortem confirmative diagnosis often detects a massive loss of SNpc DA neurons and the presence of the characteristic cytoplasmic inclusions called Lewy bodies in survived neurons. Except for a small fraction of early onset cases of PD that are linked to mutations in a dozen genes, most cases of PD are idiopathic [1, 4]. Risk factors for idiopathic PD include age, genetic predisposition, and exposure to agents such as pesticides, metals, and infectious agents [5]. 

Findings from epidemiological studies and analysis of postmortem PD brains and animal PD models have provided increasing evidence to support a role for inflammation in the brain in the pathogenesis of PD [6]. And in the process of Parkinson's disease (PD), neuroinflammation appears early and nearly persists throughout the disease course [7]. Moreover, during the early life occurrence of inflammation in the brain, as a consequence of either brain injury or exposure to infectious agents, This process may play a role in the pathogenesis of PD [8].

Microglia are the resident immune cells in the brain and have critical roles in immune surveillance under normal conditions. However, activated microglia release pro-inflammatory molecules such as IL-1*β*, tumor necrosis factor*α* (TNF-*α*), and nitric oxide (NO), and overyield of these molecules may cause neuronal death. Furthermore, the activation of microglia, as a result of the initial neuronal death, may initiate a cascade of events leading to progressive neurodegeneration [9, 10]. What is more, there may be a self-propelling cycle of inflammatory process involving brain immune cells that drives the slow yet progressive neurodegenerative process [11].

Lipopolysaccharide (LPS), an endotoxin produced by gram-negative bacteria, is a strong inducer of inflammation. Injection of LPS into the SN [12] or striatum [13] can induce inflammation in the brain and has been used as another tool to produce animal models of PD. Currently, there are no therapies for modifying the course of neurodegeneration and no biomarkers for making an early diagnosis.

Recent emerging studies suggest that axonal transport disruption and axonal degeneration precede neuronal death and may be causal to disease progression in neurodegenerative diseases [14]. There is also no question that axonal transport is important, because defects in the microtubule-based machinery that drives it can cause or contribute to a number of human neurodegenerative conditions, including spastic paraplegia, amyotrophic lateral sclerosis, and Alzheimer's, Huntington's, and Parkinson's diseases [15–17]. In PD, axonal pathology has been observed in postmortem patient brain samples and toxicity inhibits anterograde and increases retrograde axonal transport [18, 19]. However, little is known about the key pathological events of axonal transport during the inflammation preceding overt neuronal degeneration in Parkinson's disease (PD).

In the present study, we examined the expression of presynaptic and axonal transport proteins in SNpc involved in PD Prosess-process, using a single nigral injection of LPS, in order to look for a biomarker of PD and thus eventually reach the purpose of improving clinical symptoms and slowing or reversing the natural course of PD.

## 2. Materials and Methods

### 2.1. Materials

LPS (Sigma), tyrosine hydroxylase, anti-TH, anti-Ox42, anti-syntaxin, anti-synaptophysin, anti-SNAP25, anti-Vamp-2, anti-dynein, anti-dynactin, anti-Rab3A, anti-tubulin, anti-actin primary antibodies, and anti-mouse/rabbit/goat IgG secondary antibody were purchased from Santa Cruz Biotechnology (Santa Cruz, CA, USA). Enhanced chemiluminescent (ECL) substrate was from Pierce (Rockford, IL, USA). Mouse TNF-*α* and IL-1*β* ELISA kit was purchased from R&D Systems (Minneapolis, MN, USA).

### 2.2. Animals and Treatment

Male C57BL/6J mice (18–20 g) in this study were provided by the Experimental Animal Center of Chinese Academy of Medical Sciences. They were housed in a temperature and light control room (23°C, 12 h light cycle) and had free access to food and water. All animals were handled in accordance with the standards established in the Guide for the Care and Use of Laboratory Animals published by the Institute of Laboratory Animal Resources of the National Research Council (United States) and approved by the Animal Care Committee of the Peking Union Medical College and the Chinese Academy of Medical Sciences.

### 2.3. LPS Nigral Injection

Mice were randomly grouped as the vehicle group (control group, saline injection) and the LPS-injected group (model group, the mice were injected with LPS in nigral, except the vehicle group, which was given saline). Initially, mice were anesthetized with urethane chloral hydrate, and set in a stereotaxic instrument. LPS solution (2 *μ*L, 2 mg/mL) was injected into the right substantia nigra following Figure 2 (coordinates: A3.33, L1.25, H4.5 mm) at a flow rate of 1 *μ*L/min using a microinfusion pumps. The needle was left in place for 5 min after injection and withdrawn slowly. Saline was employed for vehicle injection. The mice were sacrificed for sampling the nigral and striatum at 1 d, 2 d, and 3 d and 1 w, 2 w, 3 w, and 5 w after injections, respectively.

### 2.4. ELISA Assay

Striatum and substantia nigra were homogenized in sterile PBS and then centrifuged at 12,000 rpm for 5 min at 4°C. Supernatants were assayed by TNF-*α* and IL-1*β* ELISA kit according to the procedures supplied by the manufacturer.

### 2.5. Tissue Preparation for Immunohistochemistry

Animals were terminally anesthetized with an overdose of sodium pentobarbital (100 mg/kg, i.p.) and perfused intracardially with heparinized saline (0.1% heparin in 0.9% saline) followed by paraformaldehyde (4% in PBS). The brains were removed and postfixed for 8 h in 4% paraformaldehyde solution. All immunohistochemistries were performed on randomly selected series of sections. Sections were treated for 5 min in 3% hydrogen peroxide, washed three times in PBS, and incubated in 10% normal goat serum (NGS) and 0.2% Triton X-100 in PBS (PBS-T) for 1 h before overnight incubation at 4°C with the primary antibody diluted in 10% NGS and PBS-T. The primary antibodies used were rabbit antityrosine hydroxylase (TH) (1 : 1000) and anti-Ox42 (1 : 200). For light microscopy, biotinylated secondary antibodies (1 : 200) were used, followed by incubation in streptavidin-biotin complex for 1 h at room temperature and visualized by incubation in 3,3-diaminobenzidine (DAB) solution (Zhongshan Goldenbridge Biotechnology).

### 2.6. Immunohistochemistry

Three mice chosen randomly from each group were anesthetized and perfused with 80–100 mL normal saline by left ventricle cannula and then changed into 4% paraformaldehyde (PFA). The brain was cut into four slices firstly and fixed in 4% PFA for 4 h in 4°C; the slices could be used after sinking in the fixing agents containing 20% sucrose. The brain was further cut into slices with 8 *μ*m thicknesses. The slices were washed in PBS to remove cryopreservative. The tissue was then additionally washed in PBS. The samples were placed in goat serum for 1 h at room temperature. Incubation overnight at 4°C was performed with mouse antibody anti-Ox42. After washing in PBS, the slices were washed with PBS and incubated with biotinylated goat anti-mouse IgG for 1 h at room temperature and then incubated in avidin-biotin horseradish peroxidase macromolecular complex for 1 h at room temperature. The slices were incubated briefly in DAB substrate kit (Zhongshan Goldenbridge Biotechnology) to develop color. After a final set of washes in PBS, the slices were mounted on slides, dehydrated, cleared, and coverslipped with mounting medium.

### 2.7. Western Blot Analysis

Animals were terminally anesthetized the same way described above and perfused intracardially with heparinized saline (0.1% heparin in 0.9% saline). Striatum and substantia nigra were hand-dissected on ice-cold surface, assisted by a tissue chopper. Striatal tissue samples were homogenized using hand-held polytron homogenizer in ice-cold TEVP buffer, pH 7.4 (10 mM Tris-HCl, 5 mM NaF, 1 mM Na_3_VO_4_, 1 mM EDTA, 1 mM EGTA, and 320 mM sucrose and protease inhibitors (Sigma, 1 : 100)). The homogenate was centrifuged at 12000 rpm. Protein concentrations were determined by BCA assay (Pierce). The samples were separated by 9% SDS-PAGE and then transferred to PVDF membrane. The membrane was blocked by 3% BSA and incubated with anti-synapsin, anti-snap25, anti-syntaxin, anti-synaptophysin, anti-dynein, anti-dynactin, anti-COX2, anti-Ox42, anti-tubulin, anti-Rab3A, anti-*β*-actin antibodies, then followed by horseradish peroxide (HRP)-conjugated secondary antibody, and detected with the enhanced chemiluminescence (ECL) plus detection system. The density of each band was quantified by using image analysis software Gelpro31 (Science Lab 2005 Image Guage; Fuji Film Co. Ltd., Tokyo, Japan).

### 2.8. Statistical Analysis

Data are expressed as mean ± standard deviation (SD) as indicated. Analysis of variance (ANOVA) was performed followed by Newman-Keuls posthoc test to assess the differences between the relevant control and each experimental group. *P* < 0.05 was regarded as statistically significant.

## 3. Results

### 3.1. Upexpression of OX42 and COX2 Induced by LPS in Substantia Nigra and Striatum

**Figure 1:**
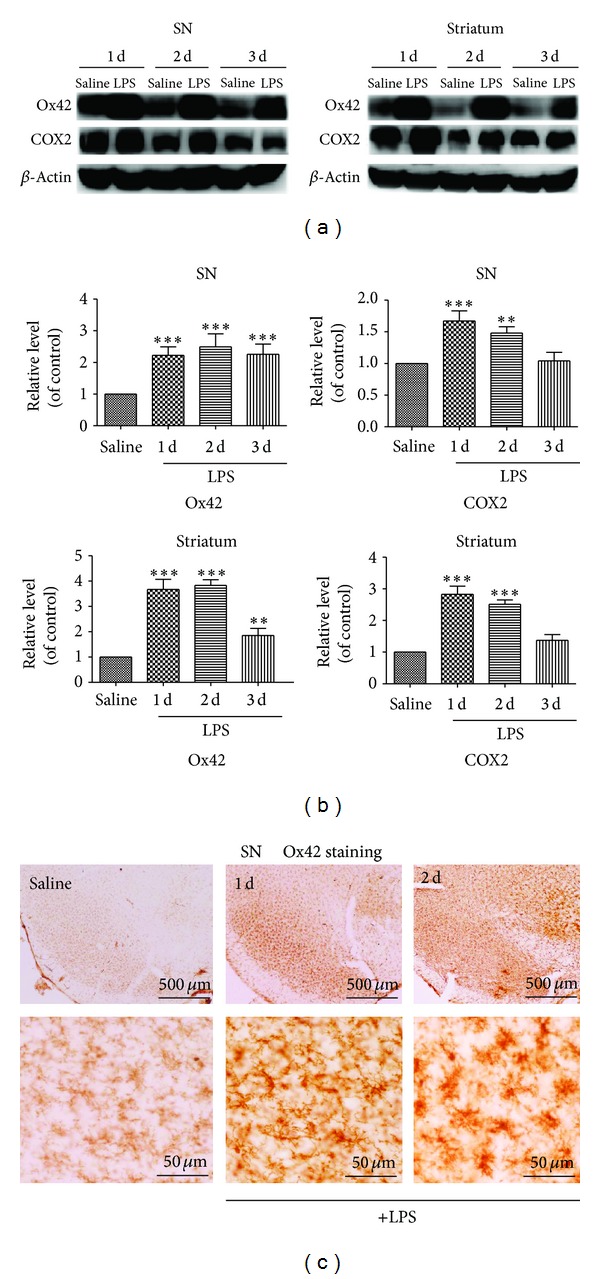
Microglia were responsing to LPS application. (a) Microglia have been significantly activated by increased levels of Ox42 in the striatum, as well as in the SN during one to three days. At the same time, the expression of COX2 increased as time passed. After mice were treated with LPS, SN and striatum were separated. The expressions of Ox42 and COX2 level were detected by Western blot analysis. LPS can cause neuroinflammation after intranigral injection. (b) Quantitative analysis of Ox42 and COX2 expression in SN and striatum during one to three days. Mean ± SD. (*n* = 3, **P < 0.01, ***P < 0.001 versus saline group). (c) The expression of Ox42 was examined by immunohistochemistry. Coronal sections of SN; immunohistochemistry staining with Ox42 antibody to visualize microglia at 1 d and 2 d after unilateral application of LPS to the pial surface or application saline on the control side.

Increased Ox42 and COX2 expression began to be detected at 24 h after LPS challenge in both the substantia nigra and striatum (Figures 1(a) and 1(b)). The increased nigral COX2 immunoreactivity was gradually reduced during 1~3 d and returned to the basic level at three days, while the increase in striatum Ox42 expression reached a peak at one day and was still prominent at three days.

As shown in Figure 1(c), the number of Ox42-immunopositive microglia increased dramatically in SN in only LPS-injected group. Injection of LPS into the nigral markedly increased the number of Ox42 positive microglia in the nigral at 1 d after injections. In saline application animal, highly ramified (presumably resting) microglia were present throughout the process. In LPS-treated SN, microglia morphology was altered from the ramified form typical of the quiescent state to a rounded amoeboid shape, with thicker proximal processes and loss of distal ramification in the first 1 to 2 d after treatment.

### 3.2. Release of TNF-*α* and IL-1*β* Induced by LPS in Substantia Nigra and Striatum

**Figure 2:**
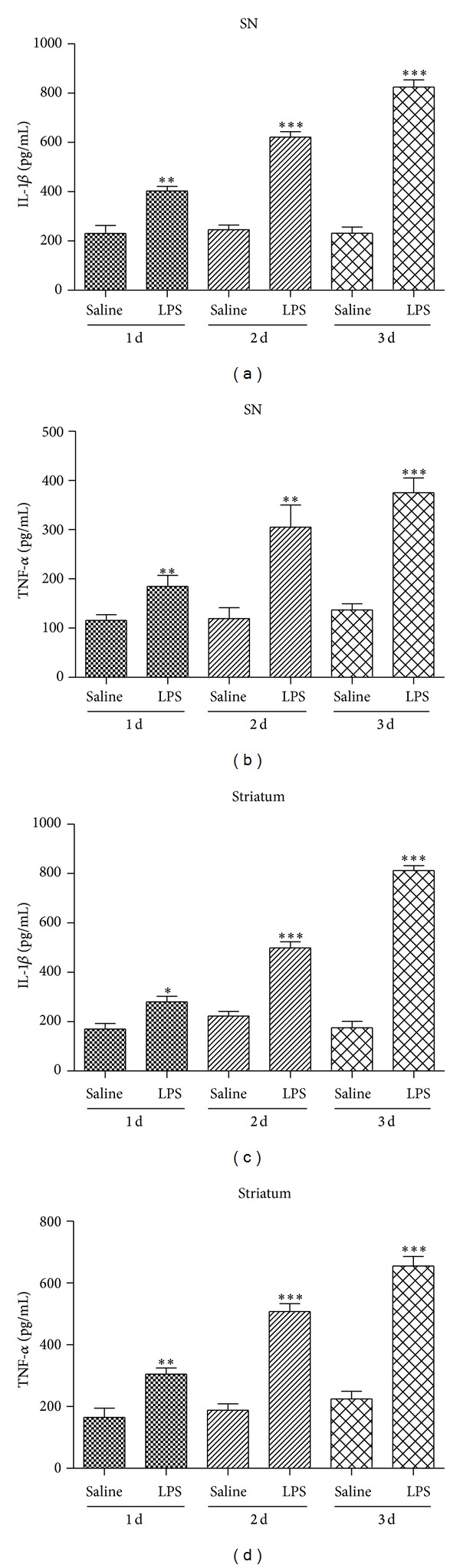
The release of TNF-*α* and IL-1*β* was increased both in the striatum (a-b) and the SN (c-d) after mice substantia nigra injection of LPS during one to three days. LPS increased TNF-*α* and IL-1*β* production in both the SN and the striatum after mice intranigral injection of LPS in a time-dependent manner. Data are shown as mean ± SD *n* = 3, *P < 0.05, **P < 0.01, ***P < 0.001 versus saline group.

The results indicated that after administration of LPS 24 h, 48 h, 72 h can elevate TNF-*α* and IL-1*β* levels of inflammatory which indicate activation of microglia. And have a time-dependent (Figures 2(a)–2(d)). In this study, intranigral injection of LPS significantly increased the release of TNF-*α* and IL-1*β* in both SN and striatum compared to that of the control group.

### 3.3. Dopaminergic Neurons Loss in Substantia Nigra

**Figure 3:**
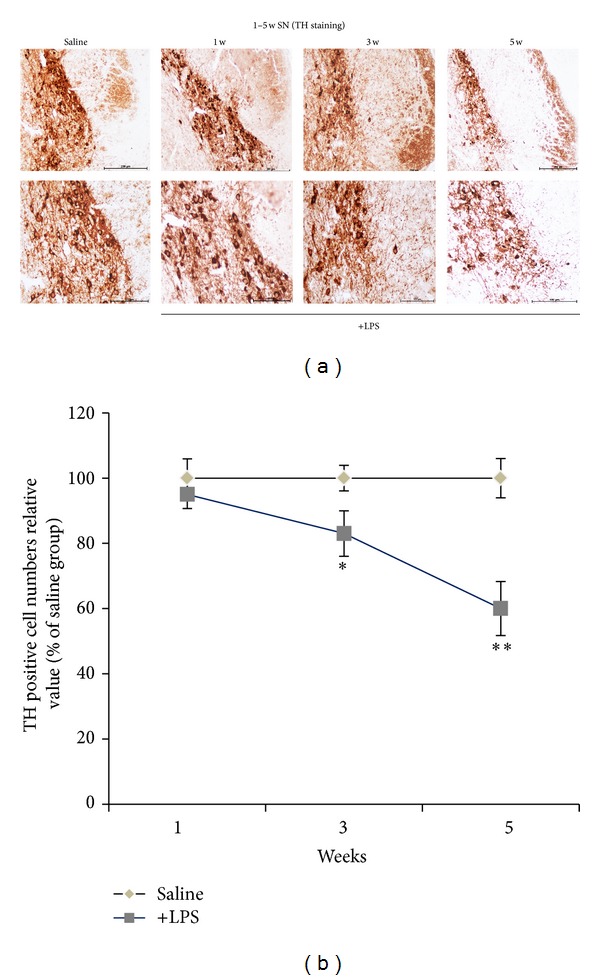
Progressive degeneration of the nigral dopaminergic neurons after intrastriatal LPS. (a) Representative TH immunochemistry staining of coronal midbrain sections demonstrates that the numbers of TH-positive neurons and fibers in the substantia nigra pars compacta are gradually reduced by intrastriatal LPS injection. Note that those TH-positive neurons in the medial substantia nigra pars compacta area are spared; scale bar: top 200 *μ*m, bottom 100 *μ*m. (b) Stereological cell counts of the TH-positive neurons in the substantia nigra pars compacta (*n* = 5-6/group, *P < 0.05, **P < 0.01 versus saline group).

After administration of LPS at 1 w, 3 w, and 5 w, animals were terminally anesthetized, and the brains were removed and postfixed for 8 h in 4% paraformaldehyde solution. TH immunochemistry staining of coronal midbrain sections was used to detect the numbers of TH-positive neurons and fibers in the substantia nigra pars compacta. The results demonstrate that the quality of TH-positive neurons has a silent reduction in 3 w; 5 w has a significant reduction (Figures 3(a) and 3(b)).

### 3.4. Dynamic Changes of Synaptic Vesicle-Associated Protein and Axonal Transport Protein in the Striatum following LPS Injection at Different Points

**Figure 4:**
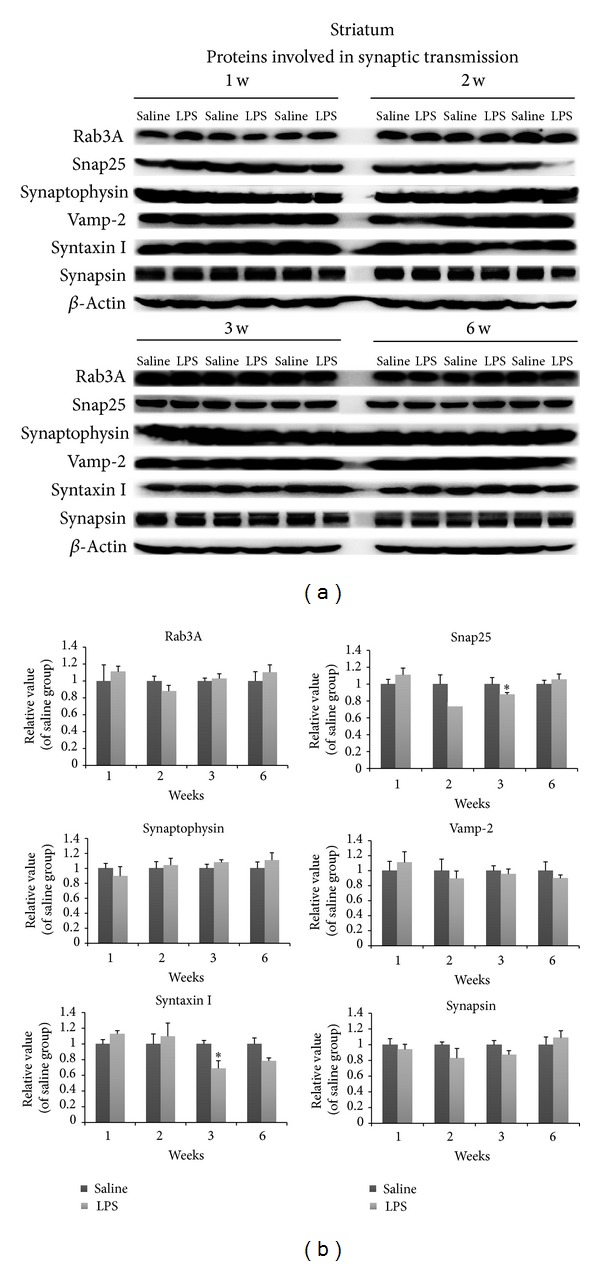
Striatal changes in the levels of proteins involved in synaptic transmission at 1 w, 2 w, 3 w, and 6 w after LPS injection. Among these proteins, snap25 levels were mildly reduced after modeling 2 w. Syntaxin I level also was reduced at 3 w. Others were not remarkably changed. (a, b) Optical densities of LPS conditions were normalized by the averaged value of saline group expressing condition. Data are shown as mean ± SD *n* = 3~6, *P < 0.05, versus saline group.

**Figure 5:**
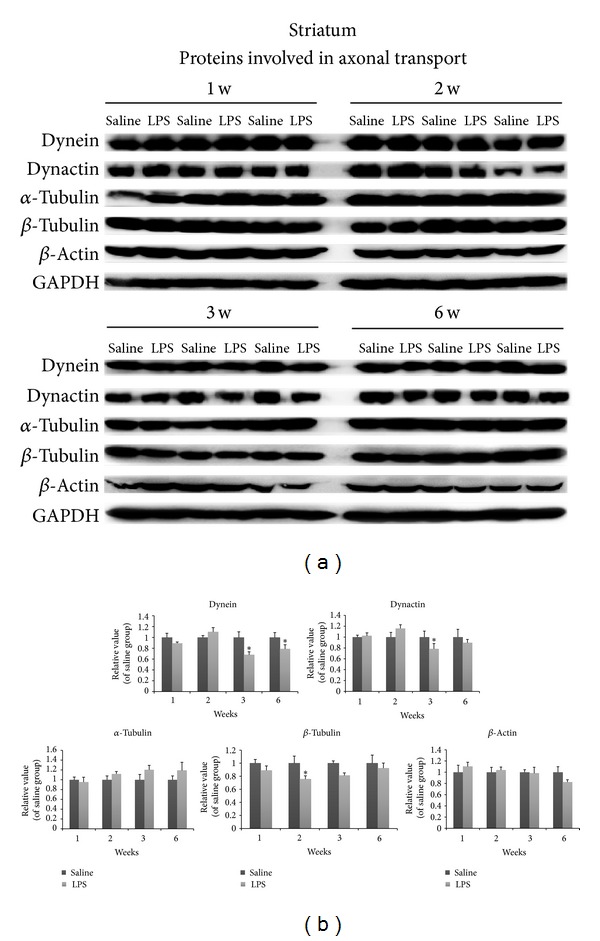
Striatal changes in proteins involved in axonal transport at different weeks after LPS injection. Levels of dynein and dynactin were reduced at 3 w. Levels of dynein were reduced at 6 w. At the same time, levels of *β*-tubulin were reduced in 2 early weeks. Optical densities of LPS injection conditions were normalized by the averaged value of saline injection. Data are shown as mean ± SD *n* = 3~6, *P < 0.05, versus saline group.

We investigated the levels of various presynaptic proteins in the striatum by Western blot analysis. At 1-2 w after LPS injection, a majority of synaptic proteins measured including synaptophysin, synapsin, Vamp-2, and Rab3A remained unchanged. However, levels of Snap25 were mildly but significantly reduced by 2 w. Syntaxin I levels were reduced at 3 w after LPS injection (Figures 4(a) and 4(b)). Since axonal transport defects have previously been shown to be causal to the type of axonal abnormalities observed, here we investigated the expression levels of transport motor and cytoskeletal proteins in the striatum after LPS injection. Levels of the retrograde transport microtubule motor protein, dynein, were significantly decreased at 3 and 6 w after LPS injection. However, while the levels of dynactin complex, which activates dynein function, were reduced at 3 w, were not altered at 6 w (Figures 5(a) and 5(b)). Among cytoskeletal proteins, *β*-tubulin levels were significantly decreased at 2 w in the striata (Figures 5(a) and 5(b)). Others were not altered after LPS injection at different weeks.

### 3.5. Dynamic Changes of Axonal Synaptic Vesicle-Associated Protein and Transport Protein in the Substantia Nigra following LPS Injection at Different Points

**Figure 6:**
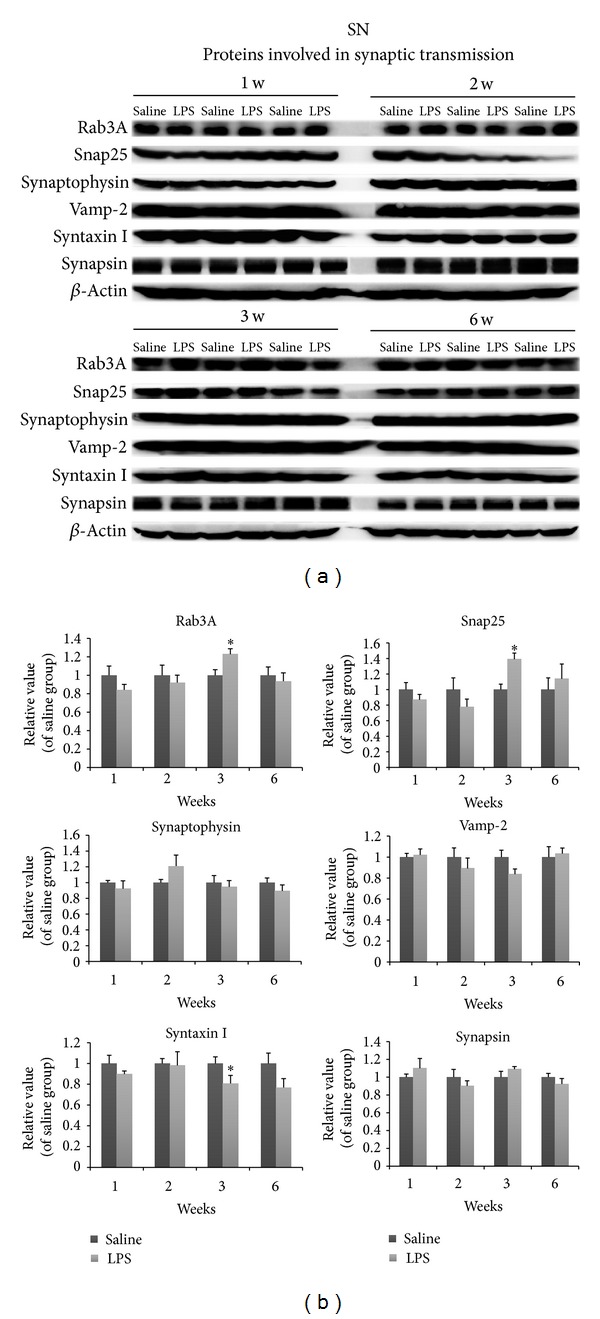
Nigral changes in the levels of proteins involved in synaptic transmission at different weeks after LPS injection. Among these proteins, Rab3A and Snap25 levels were mildly increased at 3 w, whereas syntaxin I levels were reduced. (a-b) Optical densities of LPS conditions were normalized by the averaged value of saline group expressing condition. Data are shown as mean ± SD *n* = 3~6, ^b^ *P* < 0.05, ^c^ *P* < 0.01 versus saline group.

**Figure 7:**
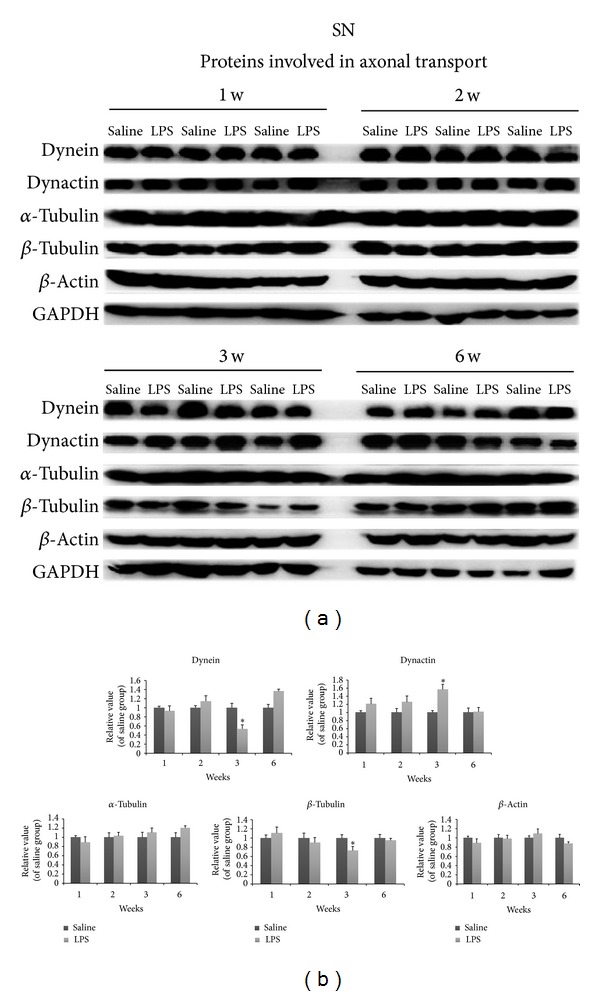
Nigral changes in proteins involved in axonal transport at different weeks after LPS injection. Levels of dynein were increased at 6 w in the SN. Levels of dynactin increased at 3 w, whereas levels of dynein and *β*-tubulin were increased at 3 w. Optical densities of LPS injection conditions were normalized by the averaged value of saline injection. Data are shown as mean ± SD *n* = 3~6, *P < 0.05, versus saline group.

The presynaptic proteins Snap25 and Rab3A both were increased at 3 w (Figures 6(a) and 6(b)). Syntaxin I levels were reduced at this time (Figures 6(a) and 6(b)). Overall, most of the synaptic proteins examined were not changed during different weeks. In contrast to presynaptic proteins, LPS nigral injection produced marked and extensive changes in the levels of transport motor and cytoskeletal proteins (Figures 7(a) and 7(b)). Dynein and *β*-tubulin showed dramatic reductions at 3 w, whereas levels of tubulin remained unchanged. Levels of retrograde transport proteins including dynactin were increased by 3 w. Dynein was increased at 6 w. Cytoskeletal proteins were dramatically altered. *β*-tubulin levels were strikingly reduced, whereas *α*-tubulin levels were not altered. In contrast to the results in the striatum, the expression levels of most of these proteins were not altered in the SN (Figures 6, 7(a), and 7(b)), suggesting that the decreased protein levels seen in the striatum were not a result of general reduction of protein production in the cell body, but most likely due to trafficking disruption.

## 4. Discussion

Research in the last two decades has unveiled an important role for neuroinflammation in Parkinson's disease (PD). Neuroinflammation is characterized by the activation of brain glial cells, primarily microglia and astrocytes, that release various soluble factors that include free radicals (reactive oxygen and nitrogen species), cytokines, and lipid metabolites [20, 21]. The majority of these glia-derived factors are proinflammatory and neurotoxic and are particularly deleterious to oxidative damage-vulnerable nigral dopaminergic neurons. As a proof of concept, the bacterial endotoxin, lipopolysaccharide (LPS), has been the most extensively utilized glial activator for the induction of inflammatory dopaminergic neurodegeneration [22]. In our research, the results indicated that after administration of LPS 24 h, 48 h, 72 h can elevate TNF-*α* and IL-1*β* levels of inflammatory which indicate activation of microglia. And have a time-dependent. Meanwhile, this process can lead to degeneration of DA neurons in 3 w after LPS injection. The quality of TH-positive neurons has a silent reduction in 3 w. At 5 w, it has a significant reduction.

A previous study has shown that disruptions in retrograde transport have been implicated in neurodegenerative disease in mice as well as in humans. Perhaps because of the long distance between cell bodies and distal axons in these neurons, sensory and motor neurons appear to be particularly sensitive to disruptions in axonal transport [23]. Rapid axonal transport is essential for neurons with long axons both for development and mature functioning. Without transport, neurotransmitter vesicles cannot move to synapses; damaged organelles in the axon cannot be replaced by healthy organelles, and target-derived survival signals cannot reach the cell body [24, 25]. Dynein is one of the major motor proteins responsible for retrograde axonal transports, that is, transport of cargo from the axons towards the cell bodies. Dynactin acts as a platform for binding dynein to its cargo and is also thought to play a role in modulation of dynein [26, 27]. Animal models disrupting the dynein-dynactin complex develop a late onset motor neuron degeneration [28], and missense mutations in a dynein subunit cause Lewy body-like inclusions and progressive motor neuron degeneration in mice [29]. Due to the central role of the dynein-dynactin complex in axonal transport, and due to evidence from animal studies, it was hypothesized that dynein changes could play a role in neurodegeneration. indeed, in our experiments, both dynein and dynactin were reduced at 3 w, and the expression of dynein is also decreased at 6 w compared with the vehicle group in the striatum. The expression of cytoskeletal protein *β*-tubulin also appears to be reduced at 2 w; the level of dynein was decreased at 3 w, but at 6 w, it has increased than the vehicle group. Dynactin has elevated changes in the opposite to the striatum at 3 w. The expression of *β*-tubulin was reduced at 3 w. Because of the importance of axonal transport, it is not surprising that disruptions in this process have been implicated in a wide range of neurodegenerative diseases, including motor and sensory neuropathies, Alzheimer's disease, Parkinson's disease, and Huntington's disease [30].

Synaptic neurotransmitter release and vesicle exocytosis include tethered vesicles from actin dissociation (stand on tethering), state (recruitment), targeted movement (targeting), raised to the presynaptic membrane anchor set (docking), vesicles start (priming), the final vesicle membrane and membrane fusion (fusion), and its contents through the fusion pore release to the process of extracellular fluid [31, 32]. This process includes a variety of neurons unique vesicle protein involved. Snap25 and syntaxin 1 are important parts of the SNARE complex the stability of the SNARE complex formation is a necessary process of the vesicle fusion. Rab3A plays a key role in the formation of complexes [33]. Our results suggest that the expression of Snap25 was decreased at 2 w in the striatum, and syntaxin I was decreased at 3 w in the substantia nigra. The expression of synaptic vesicle proteins Rab3A and Snap25 was increased at 3 w; however, the expression profile of Snap25 is contrary to the striatum. Syntaxin I is reduced at 3 weeks in line with the changes in the striatum.

These proteins reduced expression will affect the neuron transmitter release, thereby affecting the physiological functions of the neurons [34, 35]. In the substantia nigra, the expression of synaptic vesicle proteins Rab3A and Snap25 is increased at 3 w, and contrary to Snap25 expression, it changes in the striatum, indicating that the inflammation has two different influences on the substantia nigra and the striatum. The specific mechanism remains to be further studied.

In summary, the results in this study indicate that microglia activated by LPS release a variety of proinflammatory and cytotoxic factors; the process of these factors accumulation is thought to contribute to the loss of DA neurons. These results also demonstrate the changes in proteins relevant to synaptic transmission and axonal transport coupled with neuroinflammation during neuronal death. We believe that such early pathophysiological changes may provide valuable information about the mechanisms that initiate cellular dysfunction leading to degeneration. Therefore, this approach may also yield clues to biomarkers for early degenerative stages, extending a window for potential early and disease-modifying treatment of neurodegenerative diseases.
